# Semi-quantitative Analysis of EBUS Elastography as a Feasible Approach in Diagnosing Mediastinal and Hilar Lymph Nodes of Lung Cancer Patients

**DOI:** 10.1038/s41598-018-22006-4

**Published:** 2018-02-23

**Authors:** Honghai Ma, Zhou An, Pinghui Xia, Jinlin Cao, Qiqi Gao, Guoping Ren, Xing Xue, Xianhua Wang, Zhehao He, Jian Hu

**Affiliations:** 1 Department of Thoracic Surgery, the First Affiliated Hospital, School of Medicine, Zhejiang University, Hangzhou, 310003 China; 2 Department of Pathology, the First Affiliated Hospital, School of Medicine, Zhejiang University, Hangzhou, 310003 China; 3 Department of Radiology, the First Affiliated Hospital, School of Medicine, Zhejiang University, Hangzhou, 310003 China; 4 Department of Operation, Hangzhou Chinese Traditional Medicine Hospital, Hangzhou, 310003 China

## Abstract

This study aimed to semi-quantitatively evaluate the elastographic imaging color distribution of mediastinal and hilar lymph nodes (LNs), and explored its utility in helping define malignant and benign LNs for lung cancer patients. We prospectively collected patients who underwent preoperative mediastinal staging of suspected lung cancer by EBUS-TBNA. We analyzed the elastography color distribution of each LN and calculated the blue color proportion (BCP). The LN elastographic patterns were compared with the final EBUS-TBNA pathological results. A receiver operating characteristic (ROC) curve was constructed to evaluate the diagnostic value of BCP. We sampled and analyzed 79 LNs from 60 patients. The average BCP in malignant LNs was remarkably higher than that in benign LNs (57.1% versus 30.8%, *P* < 0.001). The area under the ROC curve (AUC) for the BCP was 0.86 (95% CI: 0.78–0.94). The best cutoff BCP for differentiating between benign and malignant LNs was determined as 36.7%. All the 16 LNs (20.3%) with a BCP lower than 27.9% were diagnosed as benign tissues. Our study suggests that elastography is a feasible technique that may safely help to predict LN metastasis during EBUS-TBNA. We found a clear BCP cutoff value to help define positive and negative LNs.

## Introduction

The accurate classification of lymph nodes (LNs) as benign or malignant is essential for lung cancer staging. In addition to systematic LN dissection during surgery and pathological diagnosis afterwards, there are several other minimally invasive or noninvasive methods available to detect LN metastasis before surgery, such as computed tomography (CT), endobronchial/endoscopic ultrasound transbronchial needle aspiration (EBUS/EUS-TBNA), positron emission tomography-CT (PET-CT), B-mode morphological ultrasound and elastography. Early studies applied chest CT for nodal staging, using a measurement of 1.0 cm as the threshold beyond which LNs were defined as enlarged and suspected to contain metastasis. However, the results of these studies varied, with pooled sensitivities of 57%, specificity of 82%, positive predictive value of 56% and negative predictive value of 83%^1^. PET-CT has been proven to be more efficient than CT in distinguishing benign from malignant mediastinal LNs. However, several research groups have provided evidence to indicate that PET-CT was insufficient to replace mediastinoscopy for mediastinal staging in patients with lung cancer^2,3^, as long as some controversies were unresolved^4^. In recent years EBUS-TBNA has emerged as a revolutionary method to estimate and manage both benign and malignant diseases with higher diagnostic yield, which might also replace traditional mediastinoscopy in patients with potentially resectable non-small cell lung cancer (NSCLC). Elastography is a new technique that has been utilized recently in the diagnostic procedures of focal lesions in breast, thyroid, pancreatic and prostate gland tissues^5–8^.

EUS elastography was reported to be a highly sensitive and specific method for detecting malignant involvement of pancreatic lesions and LNs. This led to utilization of elastography in EBUS. Usually, LNs containing neoplastic components are of higher cellularity and vascularity, which would cause more areas of the tissue to be relatively stiff, compared to normal ones. Yet with only some small-scale papers published to date on the topic of EBUS elastography^9–15^, the quantitative method of evaluating elastography’s role in differentiating benign and malignant mediastinal and hilar LNs is still not well delineated. We conducted this study to investigate the utility of elastography in diagnosing hilar and mediastinal LNs during EBUS-TBNA, and to identify a threshold to distinguish between benign and malignant lesions based on pathological results of EBUS-TBNA and the color distributions of elastographic images, using a newly developed semi-quantitative method.

## Results

### Patients and LN diagnostic characteristics

The baseline characteristics of all the LNs evaluated in our study were shown in Table 1. There were 60 patients (40 males, 20 females; mean age 61.6 years) who underwent elastography with EBUS-TBNA, from whom 79 hilar and mediastinal LNs were sampled and analyzed. After histological examination of the EBUS-TBNA sampled specimens, 39 malignant LNs and 40 benign LNs were diagnosed. Among the malignant LNs, there were 18 cases of lung adenocarcinoma, 2 cases of lung squamous cell carcinoma, 9 cases of small cell carcinoma, 9 cases of poorly differentiated carcinoma, and 2 cases of intestinal metastasized lung carcinoma. Among the negative LNs, diagnoses were normal lymphatic tissue (n = 22), inflammatory exudation (n = 14), bacterial infection (n = 2), and non-caseating granulomas sarcoidosis (n = 2). The sensitivity and specificity of conventional EBUS features in predicting malignant nodal metastasis were 100% and 5% for short axis size > 1 cm, 28.2% and 100% for round shape, 30.8% and 90% for distinct LN margin, 64.1% and 32.5% for heterogeneous echogenicity, and 48.7% and 50.0% for vascular pattern. There were 28 LNs with PET-CT scan images. The sensitivity and specificity of PET-CT [standardized uptake value (SUV) > 2.5] in predicting LN metastasis were 100% and 15.4%, respectively. Table 2 showed the correlation distribution of SUV value on PET-CT, EBUS elastographic types, B-mode features and vascular patterns of LNs.

**Table 1:** Baseline characteristics of the patients and lymph nodes.

LN or patients’ Characteristics	No. or median(range)		P value
BCP	<36.7%	>36.7%	
Gender	29	59	0.147
Female	13	14	
Male	16	36	
Mean age(range)	59.55	63.52	0.097
Smoking index			0.555
≤400	14	15	
>400	7	12	
Mean short axis size, mm (range)	14.8	14.6	0.876
Lymph node station	29	50	0.469
Upper paratracheal (2 R)	2	5	
Lower paratracheal (4 R, 4 L)	3	13	
Subcarinal (7)	17	22	
Hilar (10 R, 10 L)	4	8	
Interlobar(11 R, 11 L)	2	1	
Lobar(12 R, 12 L)	1	1	
Lymph node pathology			0.000
Malignant	3	36	
Lung adenocarcinoma	0	17	
Lung squamous cell carcinoma	0	2	
Lung small cell carcinoma	1	8	
Other non-small cell lung carcinoma	2	7	
Metastatasized Lung Cancer	0	2	
Benign	26	14	
Normal lymphatic tissue	16	6	
Bacterial infection	9	7	
Non-caseating granulomas sarcoidosis	1	1	
Diagnosis of primary lesions			0.158
Benign	29	59	
Malignant	18	20	
Unknown	11	30	

Data are shown as number or mean (range). BCP = blue color proportion.

Smoking index = average root number per day*years of smoking.

**Table 2:** Correlation among EBUS elastography types, B-mode features and vascular patterns of LNs.

Echogenicity	SUV value>2.5 on PET/CT	Short axis size>1 cm	Round shape	Distinct LN margin	Heterogeneous echogenicity	Blood flow with varying vessel diameters and tortuosity
BCP < 36.7%	9/26(34.6%)	28/77(36.4%)	1/11(9.1%)	2/16(12.5%)	21/52(40.4%)	15/39(38.5%)
BCP > 36.7%	17/26(65.4%)	49/77(63.6%)	10/11(90.9%)	14/16(87.5%)	31/52(59.6%)	24/39(61.5%)
Total LNs	26/79(32.9%)	77/79(97.5%)	11/79(20%)	16/79(20.3%)	52/79(65.8%)	39/79(49.4%)
Total Positive LNs	15/26(57.7%)	39/77(50.6%)	11/11(100%)	12/16(75.0%)	25/52(48.1%)	19/39(48.7%)

Data are shown as the number of LNs/total number of LNs and percentage (%), BCP, blue color proportion.

### Diagnosis Efficiency of EBUS Elastography Definition

**Figure 1:**
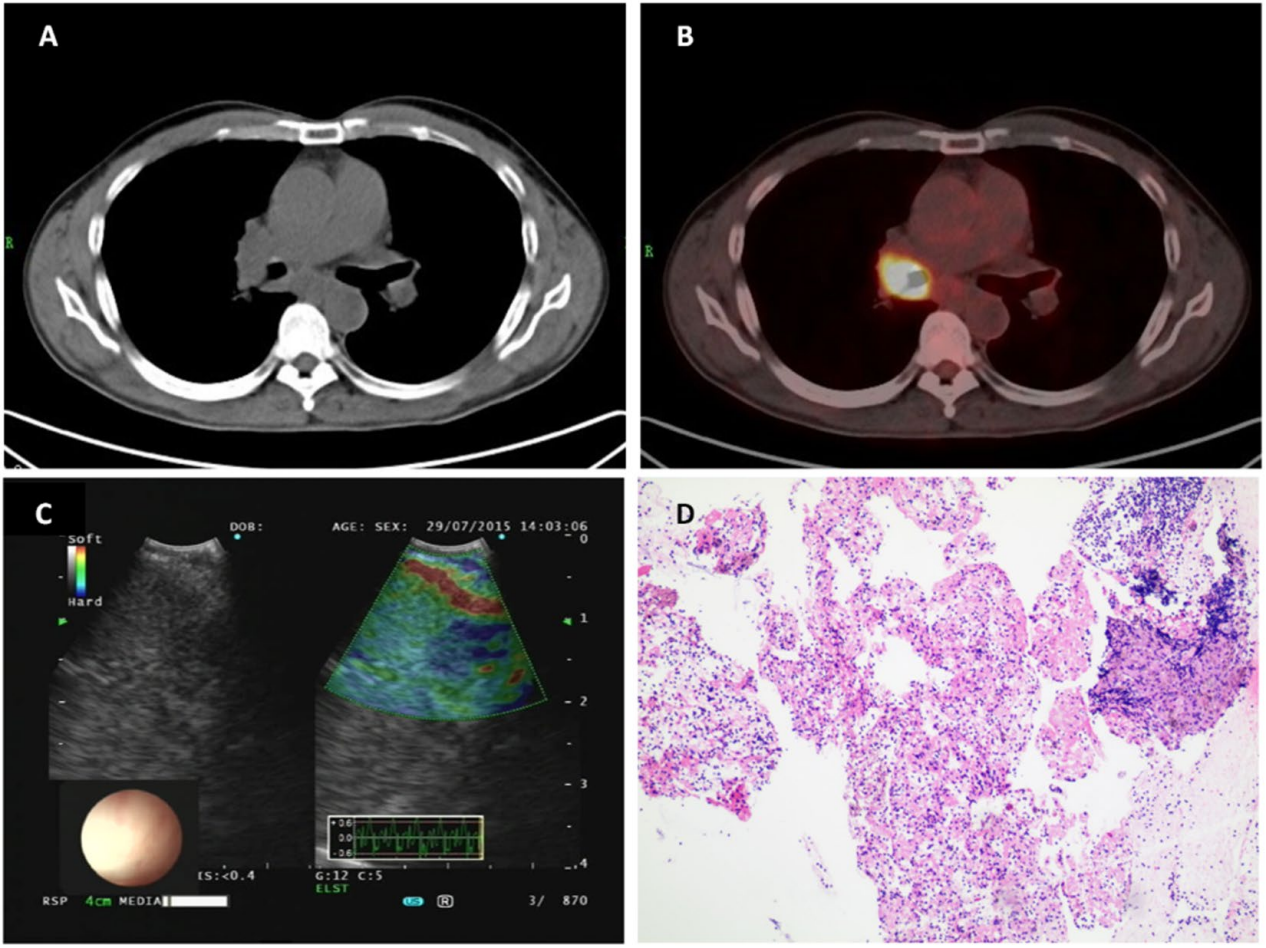
(**A**) representative case of LN with BCP < 36.7% on EBUS elastography imaging. (**A**) A subcarinal LN (#7) measuring 1.5*1.3 cm in diameter was shown on CT scan. (**B**) The FDG uptake around the area of right main bronchus, which is the location of the primary tumor, was significantly elevated on FDG-PET/CT, but no significant FDG uptake was shown in the area of the subcarinal LN (#7). (**C**) EBUS elastography showed a prodominantly non-blue (82%, mainly green) area. (**D**) Histopathological diagnosis of EBUS-TBNA specimen was normal lymphatic tissue (hematoxylin–eosin stain, 100). LN, lymph node.

**Figure 2:**
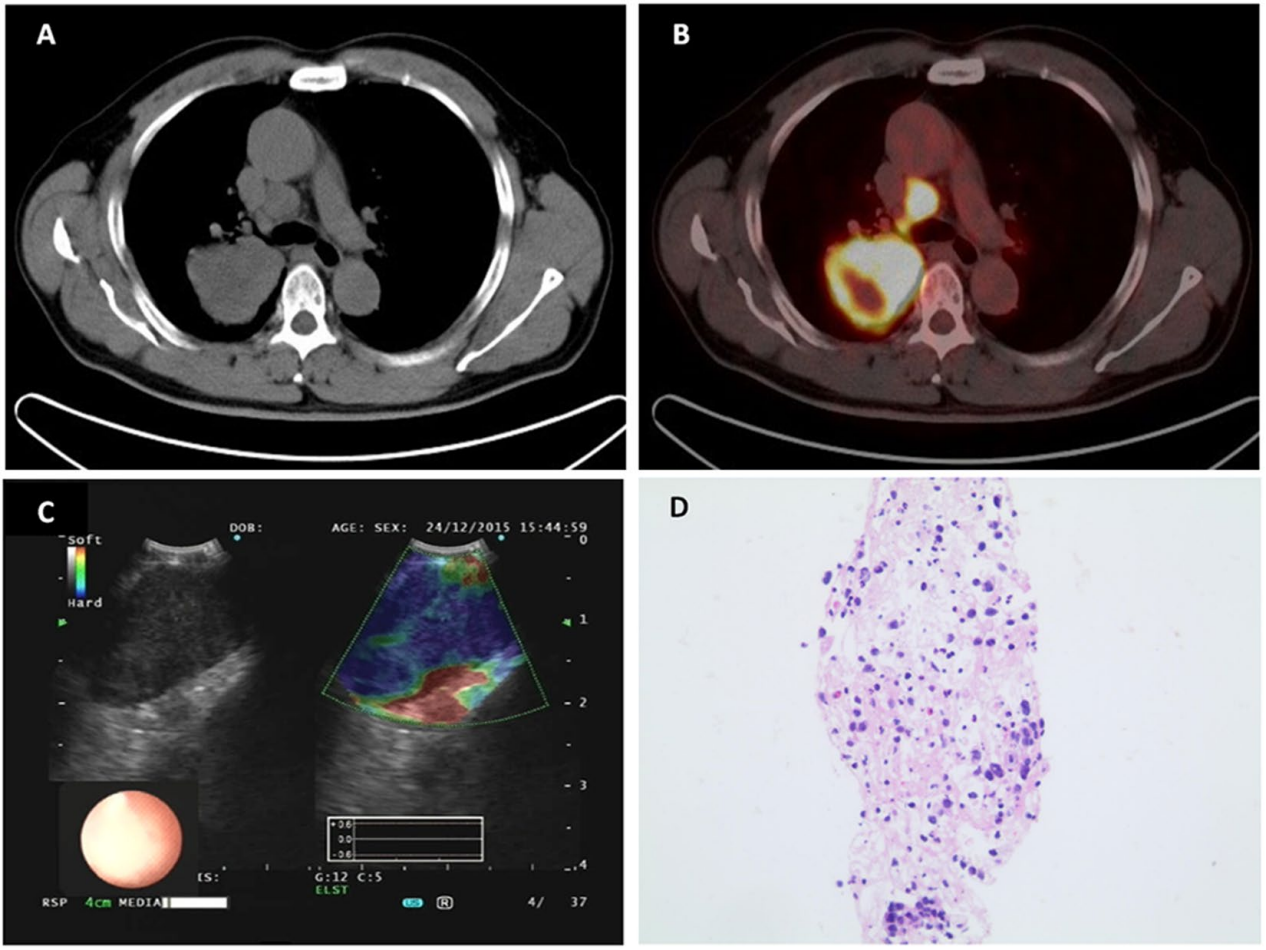
A representative case of BCP > 36.7% LN on EBUS elastography imaging. (**A**) A right lower paratracheal LN (#4 R) measuring 2.1*1.3 cm in diameter was shown on CT scan. (**B**) Significantly elevated FDG uptake in the right lower paratracheal LN (#4 R) was shown on FDG-PET/CT. (**C**) EBUS elastography imaging showed a predominantly blue (68%) area. (**D**) Histopathological diagnosis of EBUS-TBNA specimen was lung adenocarcinoma (hematoxylin–eosin stain, 200). LN, lymph node.

The AUC for the BCP was 0.86 (95% CI: 0.78–0.94). Using the Youden index (sensitivity + specificity-1), the best cutoff BCP for the differentiation of benign and malignant LNs was 36.7%. Using this cutoff value (<36.7% as benign and > 36.7% as malignant), sensitivity, specificity and accuracy were 92.3%, 67.5% and 78.5%, respectively. This cut off value showed a better efficiency compared with the above EBUS features. As shown in Table 1, the patients’ characteristics, such as smoking index, gender and age were comparable with the patients with BCP < 36.7% and BCP > 36.7%. Representative cases of EBUS elastographic imaging color distribution subtypes were shown in Figs 1 and 2. Table 3 showed the distribution of LNs according to EBUS elastographic type: with BCPs lower than the cutoff value, there were still 3 LNs diagnosed to be malignant, of which the BCPs were 27.9%, 33.1% and 32.3%. These LNs were diagnosed as two poorly differentiated NSCLC and one SCLC, respectively. In malignant LNs, the mean values of BCP were as follows: 61.9% for lung adenocarcinoma, 71.6% for lung squamous cell carcinoma, 45.8% for small cell carcinoma, 57.4% for poorly differentiated carcinoma and 50.9% for metastatic lung cancer. In benign LNs, the mean values of BCP were as follows: 27.0% for normal lymphatic tissue, 33.3% for inflammatory exudation, 49.0% bacterial infection, and 42.9% non-caseating granulomas sarcoidosis. The normal lymphatic tissue showed the lowest mean value while the lung squamous cell carcinoma showed the highest value in our patient cohort. The average BCP in malignant LNs was 57.1% (range: 27.9%–91.2%, standard deviation = 14.4), and the average BCP in benign LNs was 31.1% (range: 3.0%–62.2%, standard deviation = 17.3), which is remarkably lower (*P* < 0.001) than the malignant LNs.

**Table 3:** EBUS elastography quantification and classification of LNs.

Elastography subtype	Number of benign LNs/total LNs number (%)	Number of malignant LNs/total LNs number (%)
BCP < 36.7% (n = 29)	26/29(89.7%)	3/29(10.3%)
BCP > 36.7% (n = 50)	14/50(28.0%)	36/50(72.0%)

EBUS, endobronchial ultrasound; LNs, lymph nodes; BCP, blue color proportion.

Only six participants in the patient cohort underwent surgery, because most patients were diagnosed with late stage lung cancer, and some patients were only admitted to the outpatient department. In these six cases, four showed coincidence of diagnosis between EBUS-TBNA and a postoperative pathological diagnosis or EBUS elastography BCP results. Two cases showed a discrepant diagnosis between EBUS-TBNA and a postoperative pathological diagnosis, with the EBUS-TBNA showing negative but postoperative pathological diagnosis showing positive LNs. However, the EBUS elastography BCP results indicated that these specimens were malignant (BCP = 44.1%, finally diagnosed as poorly differentiated lung squamous carcinoma; BCP = 62.2%, finally diagnosed as poorly differentiated lung adenocarcinoma, images not shown).

### Discussion

The accurate staging of lung cancer is essential to determining appropriate treatment options. Among the different methods of preoperative staging, real-time EBUS-TBNA has been used more and more widely in clinical practice over the last decade. The recent edition of the ACCP Guidelines for Lung Cancer (3rd edition) also recommended needle-based methods as first-line approaches for invasive mediastinal staging of NSCLC^16^. Conventionally, B-mode and power Doppler imaging methods were employed to predict the possibility of cancer metastasis during EBUS scanning. Elastography is a new noninvasive EBUS modality which is hypothesized to be able to predict mediastinal and hilar nodal metastasis based on stiffness of tissue.

To the best of our knowledge, only limited published papers have evaluated elastography’s utility during EBUS^9–15^ and most of which used subjective method to classify the elastography imaging color distribution^9,11–15^. Furthermore, only one published paper gave clear correlated pathological information of detected LNs based on elastography color distributions^15^, and only a few papers involved preoperative PET-CT results^11,15^. Our data confirmed that real-time elastography during EBUS offered high sensitivity and specificity of detecting metastasis in mediastinal and hilar LNs using a firstly reported semi-quantitative method to classify the elastography color distribution. With the help of a widely used software, we were able to get the quantitative BCP and gave a clear BCP threshold to distinguish the benign and malignant LNs. Our study also provided more detailed information on disease subtypes correlated with elastography BCP. We also involved various EBUS features and SUV value in predicting malignant nodal metastasis and compared the diagnostic efficiency with our semi-quantitative method. We showed that the BCP cut off value had a better efficiency compared with the EBUS features and SUV value.

Takehiro Izumo and his research team defined the color distribution of LN elastographic imaging according to three types: Type 1, predominantly non-blue, Type 2, partly blue, partly non-blue (green, yellow and red), and Type 3, predominantly blue. The LNs that were classified as Type 1 were benign in 24/24 (100%), and Type 2 were benign in 6/14 (46.9%), while Type 3 were malignant in 35/37 (94.6%). The authors concluded that elastography is a noninvasive technique that can be performed reliably and may be helpful in the prediction of nodal metastasis during EBUS-TBNA. However, their definitions of elastography types were generally subjective and might not be replicable by other scholars. In this study, we chose a widely used software to semi-quantitatively analyze the color distribution of the LN elastography images, and the methods were easier to learn and manipulate. Rozman and his colleagues reported that when judging malignant LNs at a strain ratio ≥8, the accuracy of EBUS elastography for malignancy prediction was 86.3% (sensitivity 88.2%, specificity 84.8%, positive predictive value 81.1%, and negative predictive value 90.7%). They concluded that strain ratio was more accurate than conventional B-mode EBUS modalities for differentiating between malignant and benign LNs. However, the strain ratio was calculated by two selected round “target regions” in a LN, which might not be able to precisely predict the stiffness of the whole LN and could miss the real aspiration region of the EBUS-TBNA^10^. Our study involved a thorough evaluation of stiffness in the targeted LN and a semi-quantitative analysis of the image color distribution. Takahiro Nakajima *et al*. analyzed the color distribution of 49 LNs for 21 patients using “stiff area ratio”. Their results showed that when using a cutoff value of 0.311 for stiff area ratios, the sensitivity and specificity for predicting metastatic disease were 81% and 85% respectively. The stiff area was histologically compatible with metastatic distribution in surgically harvested LNs^17^. Our study showed similar results, but we enrolled a larger patient population (60 patients) and gave more detailed information on the color distribution of LNs with different diseases. Jiayuan Sun *et al*. utilized qualitative and quantitative methods to evaluate the ability of EBUS elastography to differentiate between benign and malignant LNs during EBUS-TBNA. The authors used a software and transformed the image to gray scale, in which the values varied from 255 (all blue pixels) to 0 (all red pixels); and the target was contoured later. The mean gray value inside the target was calculated and could reflect the stiffness of the targeted LN, which was similar to our method. The authors showed that SCLC showed a lower gray value compared to NSCLC (201.33 versus 196.37)^15^, which was similar to our result that SCLC showed a lower BCP compared to NSCLC. However, the previous study didn’t give a clear mean gray value for adenocarcinoma or squamous carcinoma subtypes, while we showed in our present study that lung squamous carcinoma had a highest BCP of 71.6%, and the average BCP for adenocarcinoma was 61.9%.

In our data, there were three cases with BCP less than 36.7% on the elastographic images, but still contained metastasis. Two of these LNs were diagnosed as SCLC, and the third was diagnosed as poorly differentiated NSCLC. We hypothesized that these tumors were generally considered to be highly aggressive, and even without a large amount of colonization they could still spread further. This may also indicate that the blue area in the LN imaging was the real area that contained metastasis, especially when the primary tumor is highly aggressive. We suggest that further research is needed to precisely examine the metastasis in the blue area of elastographic images.

Our data also showed that the BCP of LNs containing benign diseases was higher than that of normal LNs containing lymphatic tissue (33.3%, 49.0% and 42.9% versus 27.0%). These results may reveal that some diseases, such as pulmonary infection and granulomas, could lead to the elevation of LN stiffness, which may be caused by a higher density of cells and vessels. These LNs seemed to contain metastasis if assessed solely by elastography. However, compared to the benign diseases, the BCP in benign LNs was still remarkably lower than the malignant LNs (*P* < 0.001, 31.1% versus 57.1%). The pathological subtype with highest average BCP was shown to be lung squamous cell carcinoma (71.6%).

The examined LNs were diagnosed based on the results of EBUS-TBNA as the diagnostic gold standard. This design guaranteed that the examined LN area histologically corresponded to the same area examined by elastography. We also searched for postoperative pathological information for the enrolled LNs, but unfortunately these patients were mainly diagnosed as late stage and were not suitable for surgery treatment. The detection of nodal metastasis by EBUS-TBNA had a low false positive and false negative ratio^18^, but even so, a diagnostic accuracy rate exceeding 90.0% for mediastinal and hilar LNs was still convincing^19^. Therefore, the standard of EBUS-TBNA pathological diagnosis seemed to be reliable for our present investigation. As reported in previous papers, in some cases the metastatic area was localized within the LN^17^. The bronchoscopist might puncture the non-cancerous area within the LN, yielding a false negative result. To visualize the suspicious area within a LN by elastography, the bronchoscopist could control the puncture site within the LN and might improve the diagnostic efficiency of EBUS-TBNA. The limitations of this study included its retrospective, single-center nature, and our method of evaluating the BCP. In a few circumstances it was difficult to distinguish the exact edge of the LNs on elastography when it was obscured on the image.

In our practice, the average time of finishing the procedures of evaluating BCP for each LN is about 1 minute. It would be more convenient if the producer of the elastography device could provide this function in the elastography system, which could help to determine the BCP quickly during EBUS-TBNA procedure.

In summary, EBUS elastography is a useful and feasible method for predicating diagnosis of mediastinal and hilar LNs during EBUS-TBNA. It might also help to reduce the times of unnecessary biopsies of LNs and improve aspiration accuracy by taking the blue color bounded area in LNs as first priority. Further randomized, multi-center clinical studies are needed to confirm the utility of elastographic imaging during EBUS-TBNA.

## Methods

### Patient Enrollment

We enrolled into the study patients who consecutively underwent elastography during EBUS-TBNA in our department between July 29, 2015 and January 30, 2017. Each patient took a 5 mm-slice chest CT scan before their EBUS-TBNA procedure. Pre-operative PET-CT scans were also recommended for the patients, subject to financial constraints. Whenever an enlargement (10 mm in short-axis diameter) of the LN was found on a chest CT scan or an increased 18 [F]-fluorodeoxyglucose (FDG)uptake was shown [standardized uptake value (SUV) max > 2.5], both of which indicated metastasis based on the patient’s medical history and other examinations, a corresponding EBUS-TBNA was taken for the LN. Patients without a suspected primary lung cancer on PET-CT scan were excluded from this study. This study was approved by the institutional review board of the First Affiliated Hospital of Zhejiang University (reference number: 2017 intensively reviewed project, number 425). We confirmed that all EBUS-TBNA and pathological examination procedures were performed in accordance with relevant guidelines and regulations. All enrolled patients gave written informed consent before the EBUS-TBNA procedure.

### Procedure of EBUS and Determination of the Target Area

All EBUS-TBNA procedures were performed by the same bronchoscopist (Zhou An). We applied local anesthesia to the pharynx of every patient with 10 ml 4% lidocaine spray, then inserted the convex probe EBUS (CP-EBUS; BF-UC260FW, Olympus, Tokyo, Japan) through the patient’s oral route, with instillation of 2–4 ml aliquot doses of 2.0% lidocaine when needed. The bronchoscopist performed scanning at an ultrasound frequency of 7.5 MHz, and the strain elastography was performed using the ELST mode in the processor EU-ME2 PREMIER PLUS (Olympus Co., Tokyo, Japan). The enlarged target LNs on CT scan or LNs with high SUV value were identified by conventional B-mode EUBS and then elastography was performed on these LNs. Previous studies reported that LNs had the following sonographic characteristics on B-mode and vascular patterns on power Doppler indicated probable malignancy: a short-axis diameter larger than 1 cm, round shape, distinct margin, heterogeneous echogenicity and abundant blood flow with varying vessel diameters and tortuosity^20,21^. The area in a LN presenting one or more of these features was designated as the sampling area for EBUS-TBNA. When multiple LNs satisfying these criteria were found in the same station, we chose the biggest LN as the target.

### EBUS-TBNA Based Elastography Procedure

We performed elastography on each LN that was sampled for EBUS-TBNA. By comparing the elasticity of the targeted tissues with the surrounding tissues, the scanned area was reconstructed and translated into a color signal, which was then overlaid on the B-mode imaging. The colors that stood for hard, intermediate and soft tissues were blue, green and yellow/red, respectively. The complete spectrum from blue to red encoding was applied to each elastographic image and showed the calibration of relative elasticity of the scanned LN area. The B-mode and elastographic images were simultaneously displayed on the monitor side by side. The scanned area was adjusted to a maximal depth of 4.0 cm, with an aim of including the whole pre-determined targeted LN and variable portions of the surrounding tissue. After elastography evaluation, TBNA with negative pressure was done 10–20 times in the blue color abundant area of the targeted LN, under real-time EBUS guidance. The corresponding histology and cytology specimens were collected. The final diagnosis of EBUS-TBNA sampled specimens was done by pathologists who did not know the results of the EBUS elastography, and the specimens were usually viewed about 1 day after the EBUS-TBNA procedure.

### Elastographic Image Analysis

**Figure 3:**
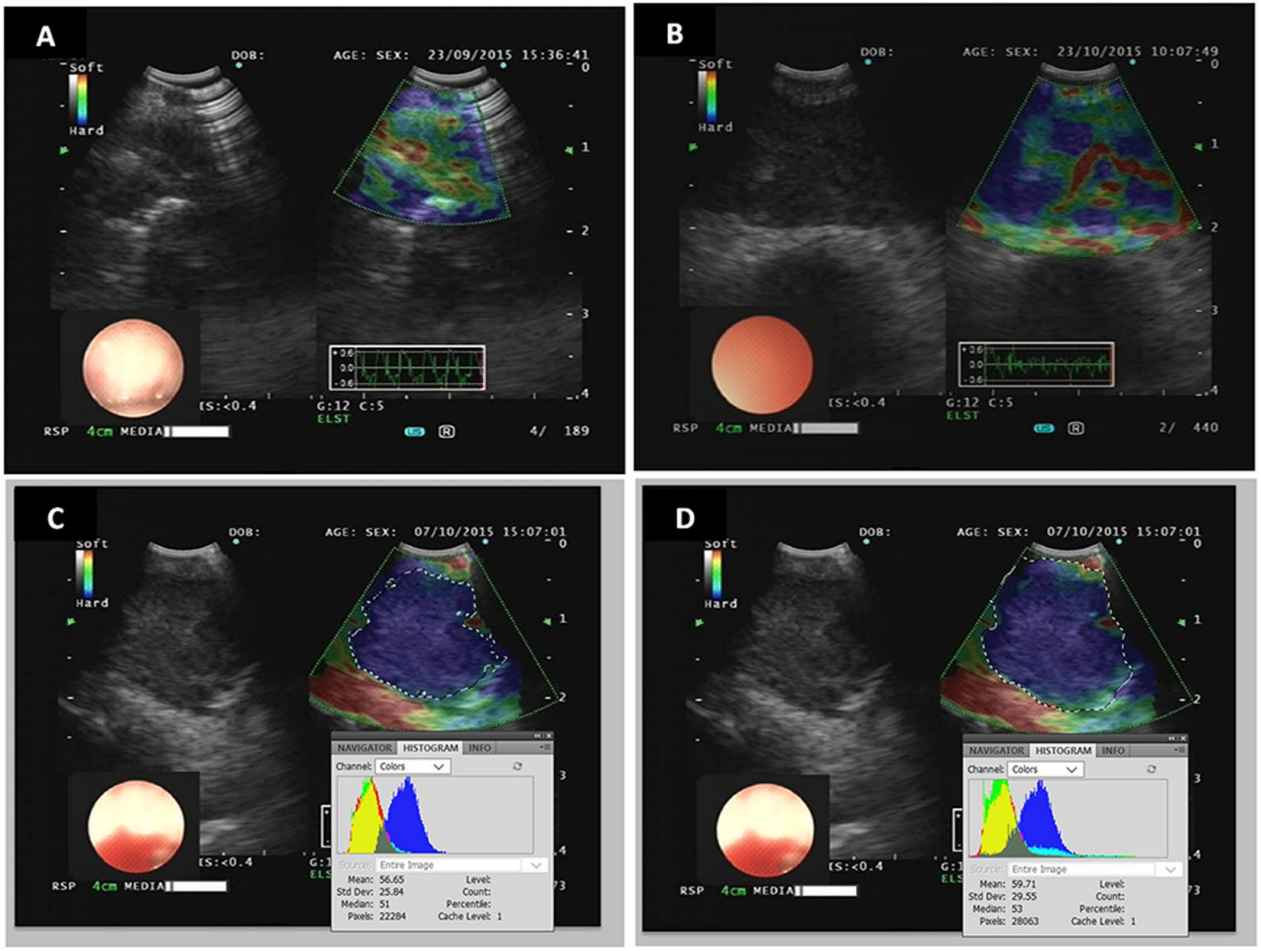
The representative color patterns of LNs on EBUS elastography imagings. For every elastogrpahy imaging like picture A, the LN was shown both by B mode ultrasound on the left and corresponding elastography imaging on the right. In the elastography image, the blue, green and red color indicated the hardest, medium and softest tissues respectively. (**A**) LN with BCP <36.7%. (**B**) LN with BCP > 36.7%. (**C**) Photoshop showed the pixels of blue color in another LN, (**D**) (same patient as **C**): Photoshop showed the pixels of the whole LN area. BCP was calculated by dividing the pixels of blue color in picture **C** with that in picture **D**. EBUS, endobronchial ultrasound; LN, lymph node.

We collected all images of the sampled LNs after every EBUS-TBNA procedure. We analyzed the color distribution of LNs, and used Photoshop (CS5 V12.0, Adobe Systems Incorporated, San Jose, CA) for the image processing which was similar to previous studies^15,22^. The blue color proportion (BCP) in the targeted LN was calculated by dividing the pixel value of the blue area by the pixel value of the whole LN. To achieve this, we used the following image processing steps in Photoshop: (1) open the “Histogram” under the “window” menu bar option, and open the raw and binary image of a LN, (2) the borders of a whole targeted LN were manually selected with “quick selection tool”, then the pixel value was shown in the “Histogram” and recorded in an Excel data sheet, (3) the blue area of the same LN was selected using the “magic wand tool”, and the pixel value was also recorded from the “Histogram” window, and (4) the BCP of this LN was calculated by dividing the pixel value of the blue area by that of the whole LN. Example images of the above processing procedures were shown in Fig. 3. In cases with multiple images, we calculated the mean BCP for each image. The elastographic patterns were compared with the final pathologic results from EBUS-TBNA.

### Data Analysis

Statistical analysis was performed with SPSS 20.0 software (Version 20.0, SPSS Inc., Chicago, IL). We constructed a receiver operating characteristic (ROC) curve and the area under the ROC curve (AUC) with 95% CI was determined to define the cutoff BCP for differentiating between benign and malignant LNs. The sensitivity and specificity of the cutoff BCP in diagnosing malignant or benign LNs were calculated separately with the final diagnosis of LNs. All statistical tests were two-sided and P < 0.05 was considered statistically significant.

### Data Availability

The datasets generated during and/or analysed during the current study are available from the corresponding author on reasonable request.
